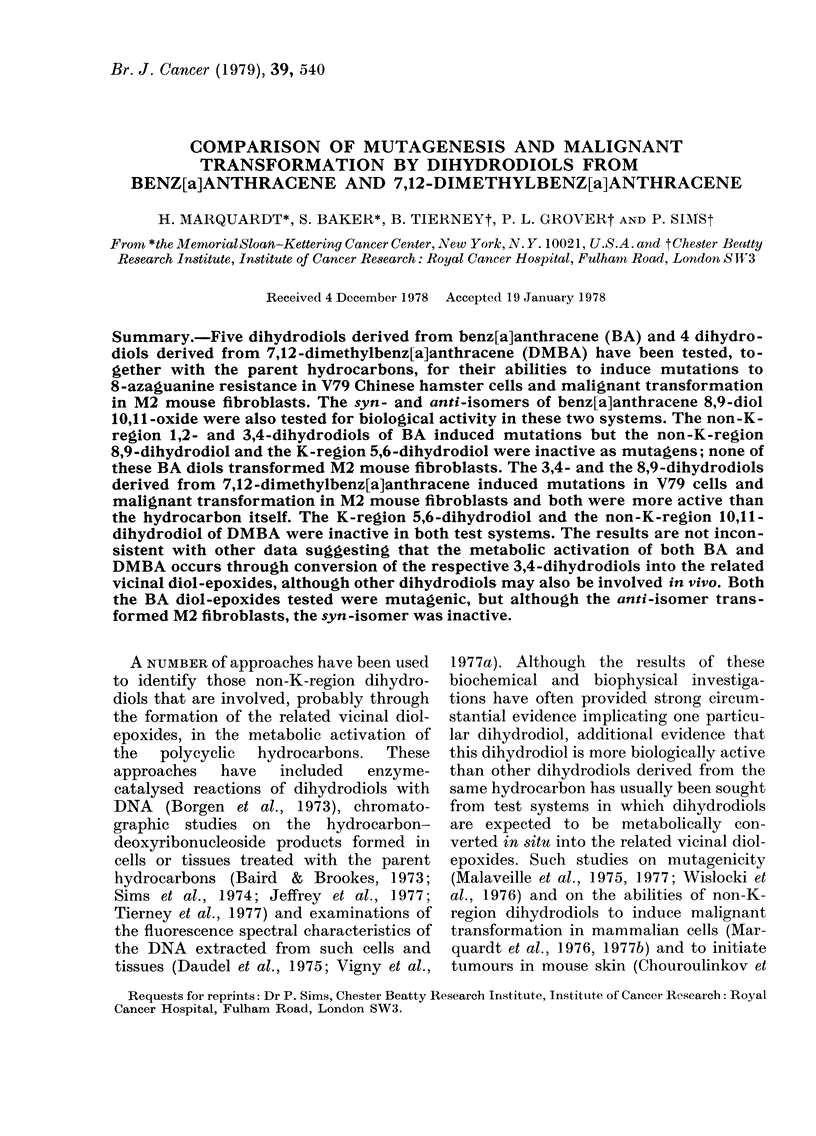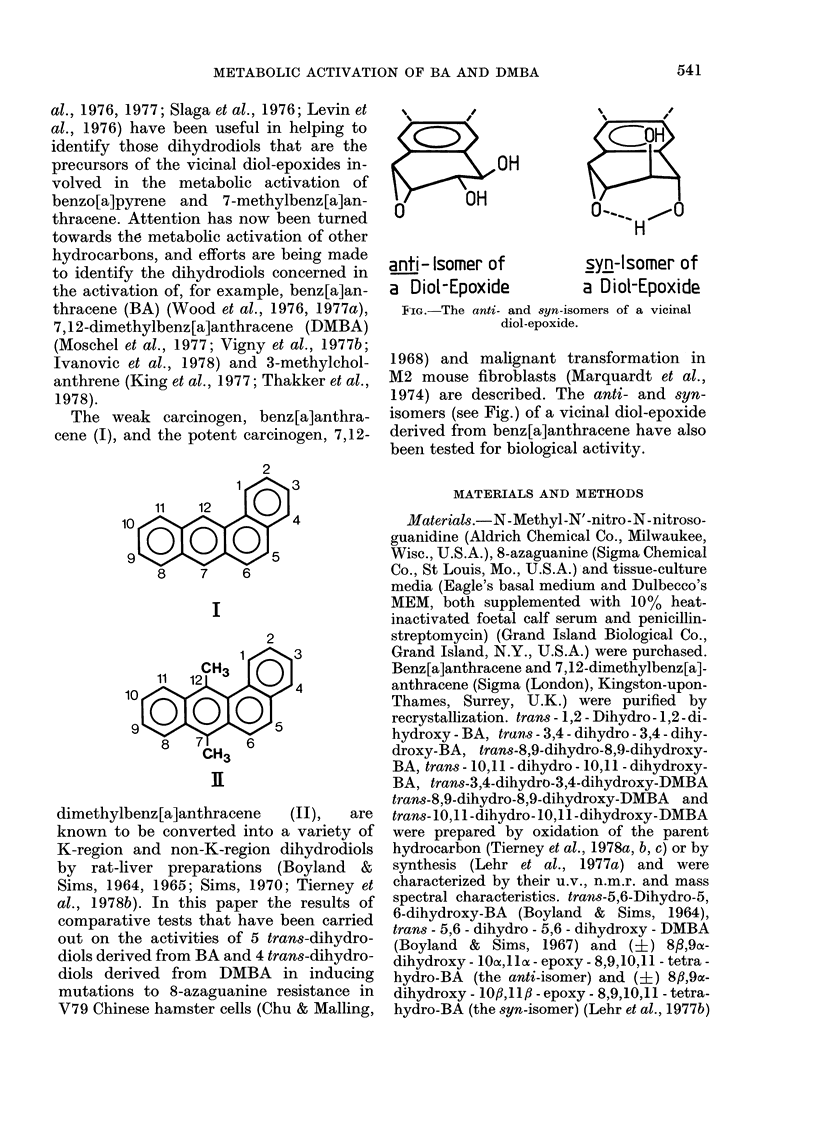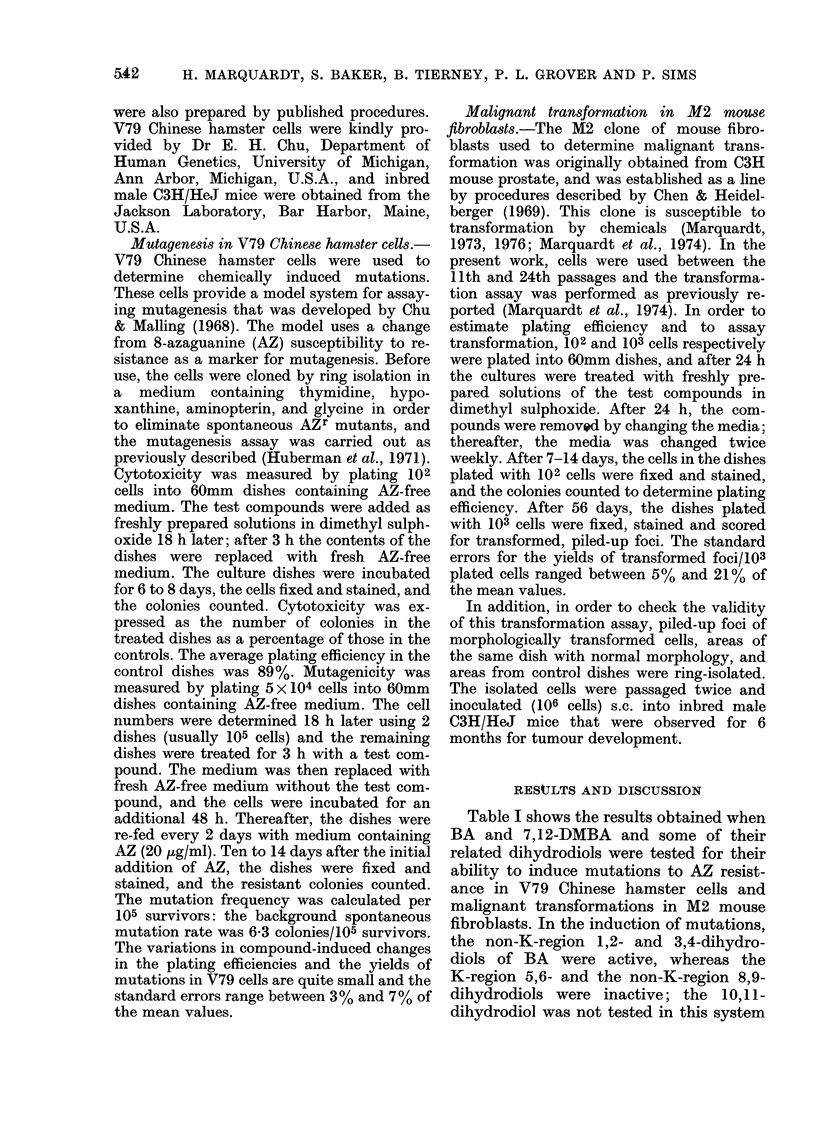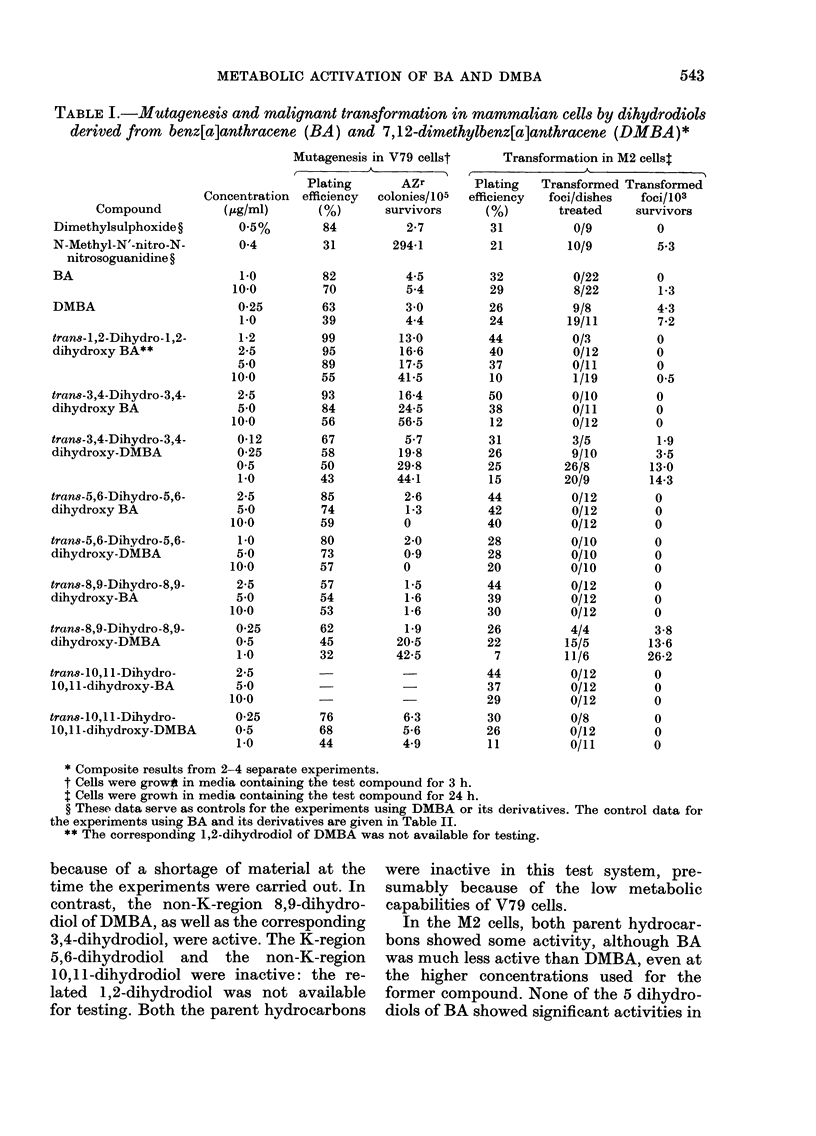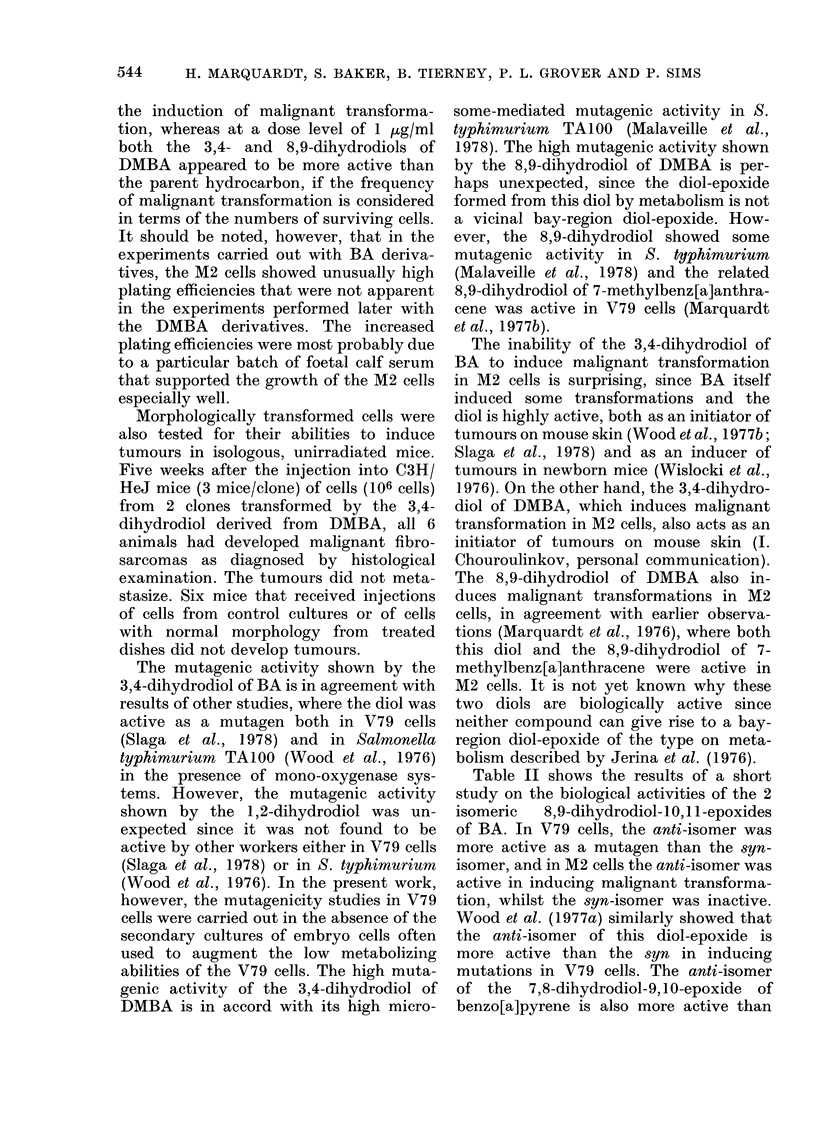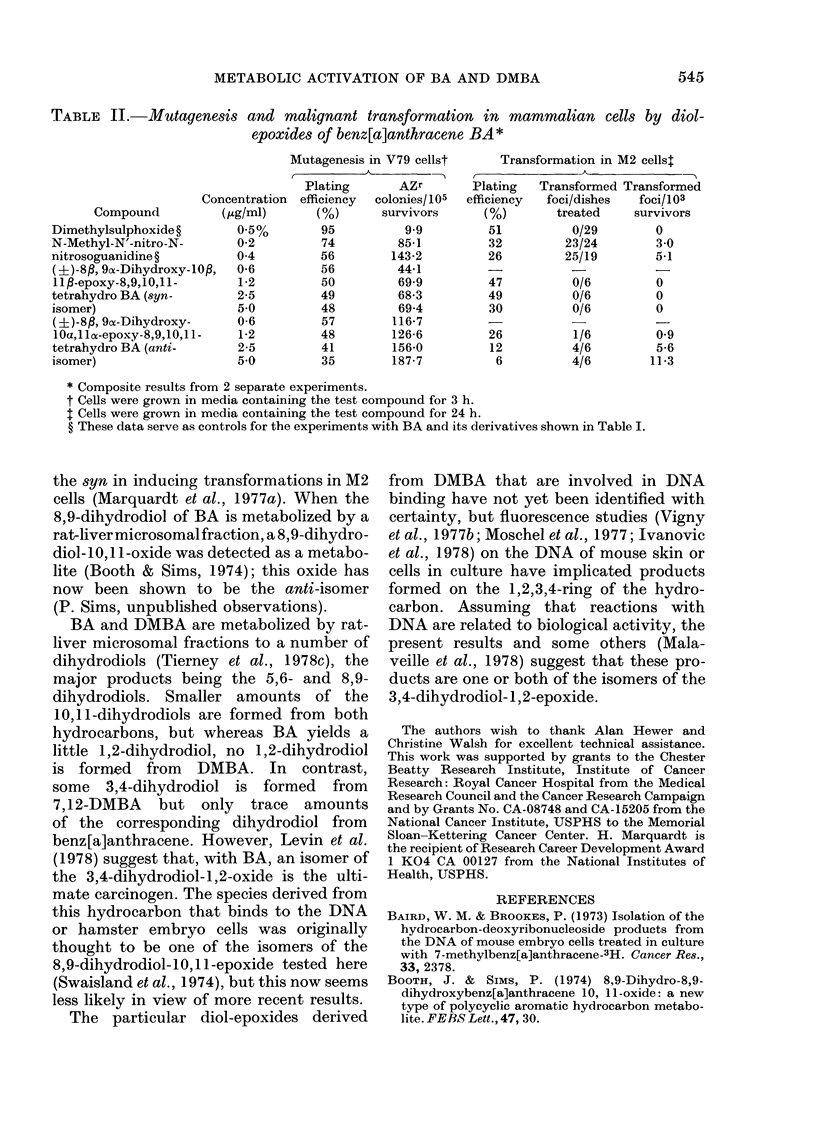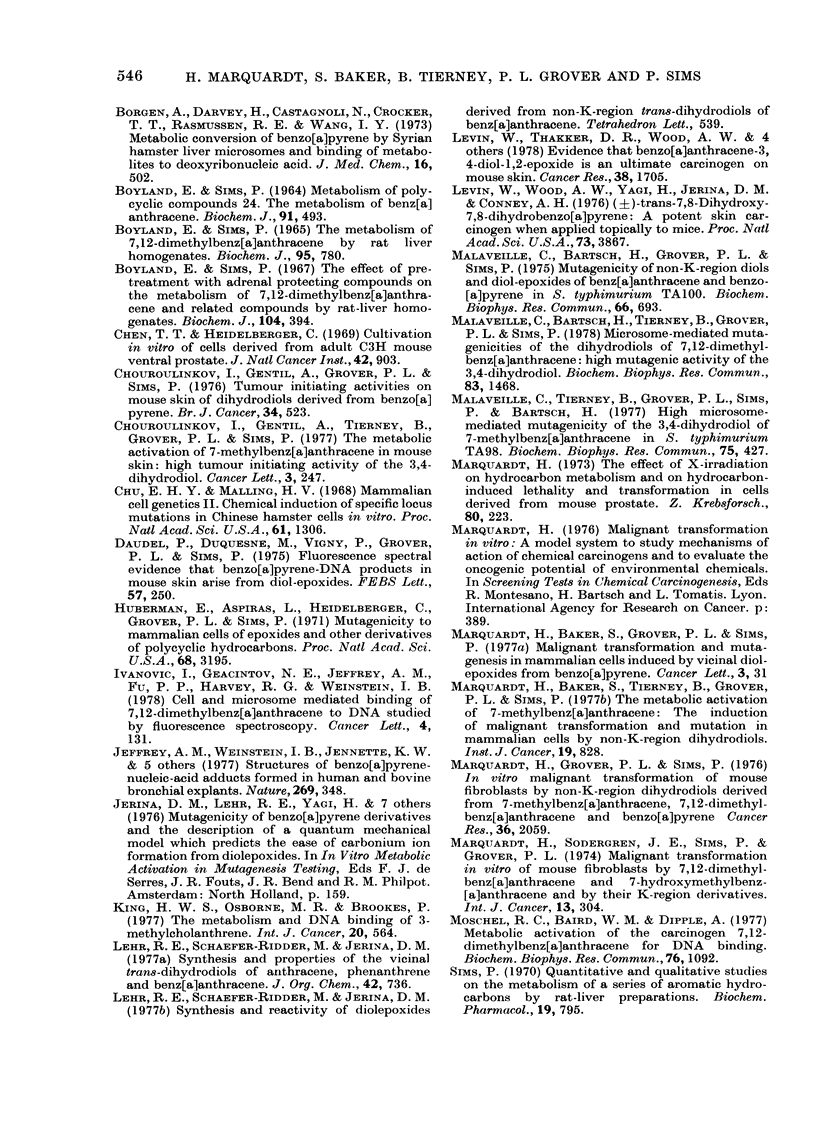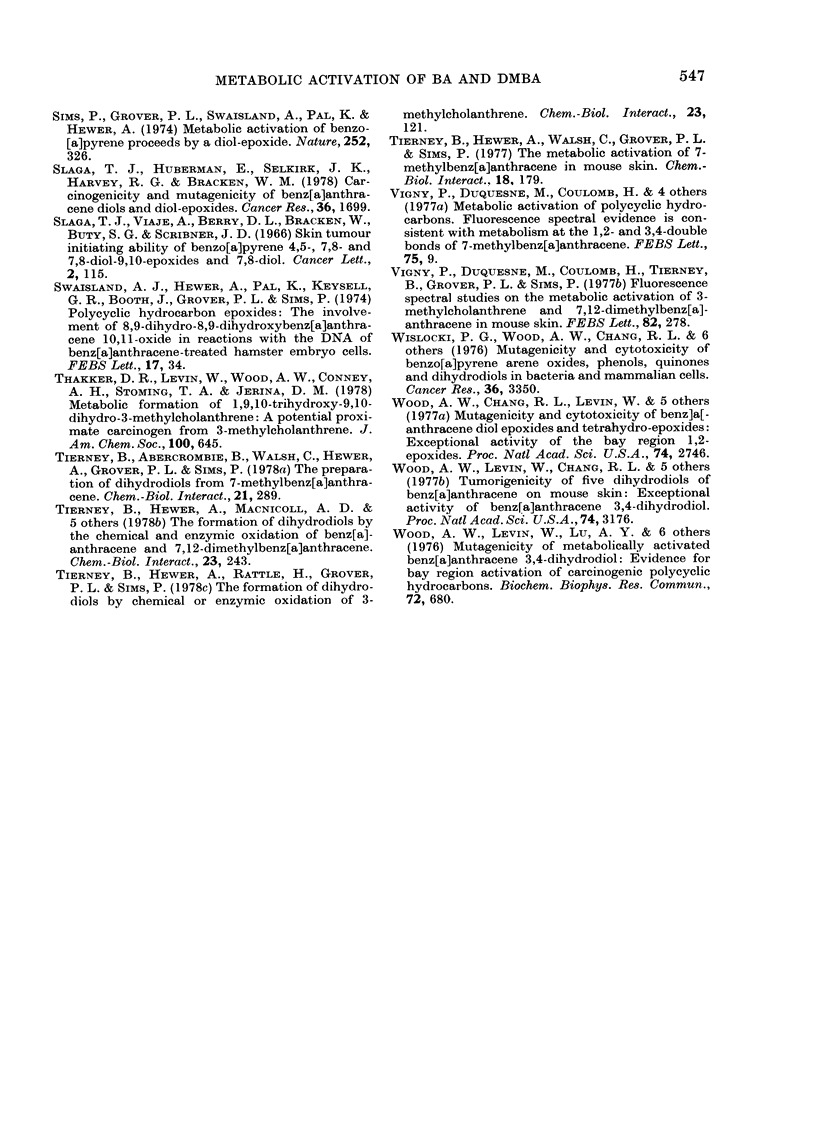# Comparison of mutagenesis and malignant transformation by dihydrodiols from benz[a]anthracene and 7,12-dimethylbenz[a]anthracene.

**DOI:** 10.1038/bjc.1979.99

**Published:** 1979-05

**Authors:** H. Marquardt, S. Baker, B. Tierney, P. L. Grover, P. Sims

## Abstract

Five dihydrodiols derived from benz[a]anthracene (BA) and 4 dihydrodiols derived from 7,12-dimethylbenz[a]anthracene (DMBA) have been tested, together with the parent hydrocarbons, for their abilities to induce mutations to 8-azaguanine resistance in V79 (Chinese hamster cells and malignant transformation in M2 mouse fibroblasts. The syn- and anti-isomers of benz[a]anthracene 8,9-diol 10,11-oxide were also tested for biological activity in these two systems. The non-K-region 1,2- and 3,4-dihydrodiols of BA induced mutations but the non-K-region 8,9-dihydrodiol and the K-region 5,6-dihydrodiol were inactive as mutagens; none of these BA diols transformed M2 mouse fibroblasts. The 3,4- and the 8,9-dihydrodiols derived from 7,12-dimethylbenz[a]anthracene induced mutations in V79 cells and malignant transformation in M2 mouse fibroblasts and both were more active than the hydrocarbon itself. The K-region 5,6-dihydrodiol and the non-K-region 10,11-dihydrodiol of DMBA were inactive in both test systems. The results are not inconsistent with other data suggesting that the metabolic activation of both BA and DMBA occurs through conversion of the respective 3,4-dihydrodiols into the related vicinal diol-epoxides, although other dihydrodiols may also be involved in vivo. Both the BA diol-epoxides tested were mutagenic, but although the anti-isomer transformed M2 fibroblasts, the syn-isomer was inactive.


*Br. J. Cancer* (1979), **39**, 540

### COMPARISON OF MUTAGENESIS AND MALIGNANT TRANSFORMATION BY DIHYDRODIOLS FROM BENZ[a]ANTHRACENE AND 7,12-DIMETHYLBENZ[a]ANTHRACENE

H. MARQUARDT*, S. BAKER*, B. TIERNEY†, P. L. GROVER† AND P. SIMS†

*From *the Memorial Sloan–Kettering Cancer Center, New York, N.Y. 10021, U.S.A. and †Chester Beatty Research Institute, Institute of Cancer Research: Royal Cancer Hospital, Fulham Road, London SW3*

Received 4 December 1978 Accepted 19 January 1978

**Summary.—Five dihydrodiols derived from benz[a]anthracene (BA) and 4 dihydrodiols derived from 7,12-dimethylbenz[a]anthracene (DMBA) have been tested, together with the parent hydrocarbons, for their abilities to induce mutations to 8-azaguanine resistance in V79 Chinese hamster cells and malignant transformation in M2 mouse fibroblasts. The *syn*- and *anti*-isomers of benz[a]anthracene 8,9-diol 10,11-oxide were also tested for biological activity in these two systems. The non-K-region 1,2- and 3,4-dihydrodiols of BA induced mutations but the non-K-region 8,9-dihydrodiol and the K-region 5,6-dihydrodiol were inactive as mutagens; none of these BA diols transformed M2 mouse fibroblasts. The 3,4- and the 8,9-dihydrodiols derived from 7,12-dimethylbenz[a]anthracene induced mutations in V79 cells and malignant transformation in M2 mouse fibroblasts and both were more active than the hydrocarbon itself. The K-region 5,6-dihydrodiol and the non-K-region 10,11-dihydrodiol of DMBA were inactive in both test systems. The results are not inconsistent with other data suggesting that the metabolic activation of both BA and DMBA occurs through conversion of the respective 3,4-dihydrodiols into the related vicinal diol-epoxides, although other dihydrodiols may also be involved *in vivo*. Both the BA diol-epoxides tested were mutagenic, but although the *anti*-isomer transformed M2 fibroblasts, the *syn*-isomer was inactive.**

A NUMBER of approaches have been used to identify those non-K-region dihydrodiols that are involved, probably through the formation of the related vicinal diol-epoxides, in the metabolic activation of the polycyclic hydrocarbons. These approaches have included enzyme-catalysed reactions of dihydrodiols with DNA (Borgen *et al.*, 1973), chromatographic studies on the hydrocarbon–deoxyribonucleoside products formed in cells or tissues treated with the parent hydrocarbons (Baird & Brookes, 1973; Sims *et al.*, 1974; Jeffrey *et al.*, 1977; Tierney *et al.*, 1977) and examinations of the fluorescence spectral characteristics of the DNA extracted from such cells and tissues (Daudel *et al.*, 1975; Vigny *et al.*, 1977*a*). Although the results of these biochemical and biophysical investigations have often provided strong circumstantial evidence implicating one particular dihydrodiol, additional evidence that this dihydrodiol is more biologically active than other dihydrodiols derived from the same hydrocarbon has usually been sought from test systems in which dihydrodiols are expected to be metabolically converted *in situ* into the related vicinal diol-epoxides. Such studies on mutagenicity (Malaveille *et al.*, 1975, 1977; Wislocki *et al.*, 1976) and on the abilities of non-K-region dihydrodiols to induce malignant transformation in mammalian cells (Marquardt *et al.*, 1976, 1977*b*) and to initiate tumours in mouse skin (Chouroulinkov *et

Requests for reprints: Dr P. Sims, Chester Beatty Research Institute, Institute of Cancer Research: Royal Cancer Hospital, Fulham Road, London SW3.

*al.*, 1976, 1977; Slaga *et al.*, 1976; Levin *et al.*, 1976) have been useful in helping to identify those dihydrodiols that are the precursors of the vicinal diol-epoxides involved in the metabolic activation of benzo[a]pyrene and 7-methylbenz[a]anthracene. Attention has now been turned towards the metabolic activation of other hydrocarbons, and efforts are being made to identify the dihydrodiols concerned in the activation of, for example, benz[a]anthracene (BA) (Wood *et al.*, 1976, 1977*a*), 7,12-dimethylbenz[a]anthracene (DMBA) (Moschel *et al.*, 1977; Vigny *et al.*, 1977*b*; Ivanovic *et al.*, 1978) and 3-methylcholanthrene (King *et al.*, 1977; Thakker *et al.*, 1978).

The weak carcinogen, benz[a]anthracene (I), and the potent carcinogen, 7,12-

I

II

dimethylbenz[a]anthracene (II), are known to be converted into a variety of K-region and non-K-region dihydrodiols by rat-liver preparations (Boyland & Sims, 1964, 1965; Sims, 1970; Tierney *et al.*, 1978*b*). In this paper the results of comparative tests that have been carried out on the activities of 5 *trans*-dihydrodiols derived from BA and 4 *trans*-dihydrodiols derived from DMBA in inducing mutations to 8-azaguanine resistance in V79 Chinese hamster cells (Chu & Malling,

FIG.—The *anti*- and *syn*-isomers of a vicinal diol-epoxide.

1968) and malignant transformation in M2 mouse fibroblasts (Marquardt *et al.*, 1974) are described. The *anti*- and *syn*-isomers (see Fig.) of a vicinal diol-epoxide derived from benz[a]anthracene have also been tested for biological activity.

### MATERIALS AND METHODS

*Materials.*—N-Methyl-N′-nitro-N-nitrosoguanidine (Aldrich Chemical Co., Milwaukee, Wisc., U.S.A.), 8-azaguanine (Sigma Chemical Co., St Louis, Mo., U.S.A.) and tissue-culture media (Eagle's basal medium and Dulbecco's MEM, both supplemented with 10% heat-inactivated foetal calf serum and penicillin-streptomycin) (Grand Island Biological Co., Grand Island, N.Y., U.S.A.) were purchased. Benz[a]anthracene and 7,12-dimethylbenz[a]anthracene (Sigma (London), Kingston-upon-Thames, Surrey, U.K.) were purified by recrystallization. *trans*-1,2-Dihydro-1,2-dihydroxy-BA, *trans*-3,4-dihydro-3,4-dihydroxy-BA, *trans*-8,9-dihydro-8,9-dihydroxy-BA, *trans*-10,11-dihydro-10,11-dihydroxy-BA, *trans*-3,4-dihydro-3,4-dihydroxy-DMBA *trans*-8,9-dihydro-8,9-dihydroxy-DMBA and *trans*-10,11-dihydro-10,11-dihydroxy-DMBA were prepared by oxidation of the parent hydrocarbon (Tierney *et al.*, 1978*a*, *b*, *c*) or by synthesis (Lehr *et al.*, 1977*a*) and were characterized by their u.v., n.m.r. and mass spectral characteristics. *trans*-5,6-Dihydro-5,6-dihydroxy-BA (Boyland & Sims, 1964), *trans*-5,6-dihydro-5,6-dihydroxy-DMBA (Boyland & Sims, 1967) and (±) 8β,9α-dihydroxy-10α,11α-epoxy-8,9,10,11-tetrahydro-BA (the *anti*-isomer) and (±) 8β,9α-dihydroxy-10β,11β-epoxy-8,9,10,11-tetrahydro-BA (the *syn*-isomer) (Lehr *et al.*, 1977*b*)

were also prepared by published procedures. V79 Chinese hamster cells were kindly provided by Dr E. H. Chu, Department of Human Genetics, University of Michigan, Ann Arbor, Michigan, U.S.A., and inbred male C3H/HeJ mice were obtained from the Jackson Laboratory, Bar Harbor, Maine, U.S.A.

*Mutagenesis in* V79 *Chinese hamster cells.*—V79 Chinese hamster cells were used to determine chemically induced mutations. These cells provide a model system for assaying mutagenesis that was developed by Chu & Malling (1968). The model uses a change from 8-azaguanine (AZ) susceptibility to resistance as a marker for mutagenesis. Before use, the cells were cloned by ring isolation in a medium containing thymidine, hypoxanthine, aminopterin, and glycine in order to eliminate spontaneous $AZ^r$ mutants, and the mutagenesis assay was carried out as previously described (Huberman *et al.*, 1971). Cytotoxicity was measured by plating $10^2$ cells into 60mm dishes containing AZ-free medium. The test compounds were added as freshly prepared solutions in dimethyl sulphoxide 18 h later; after 3 h the contents of the dishes were replaced with fresh AZ-free medium. The culture dishes were incubated for 6 to 8 days, the cells fixed and stained, and the colonies counted. Cytotoxicity was expressed as the number of colonies in the treated dishes as a percentage of those in the controls. The average plating efficiency in the control dishes was 89%. Mutagenicity was measured by plating $5 \times 10^4$ cells into 60mm dishes containing AZ-free medium. The cell numbers were determined 18 h later using 2 dishes (usually $10^5$ cells) and the remaining dishes were treated for 3 h with a test compound. The medium was then replaced with fresh AZ-free medium without the test compound, and the cells were incubated for an additional 48 h. Thereafter, the dishes were re-fed every 2 days with medium containing AZ (20 $\mu$g/ml). Ten to 14 days after the initial addition of AZ, the dishes were fixed and stained, and the resistant colonies counted. The mutation frequency was calculated per $10^5$ survivors: the background spontaneous mutation rate was 6·3 colonies/$10^5$ survivors. The variations in compound-induced changes in the plating efficiencies and the yields of mutations in V79 cells are quite small and the standard errors range between 3% and 7% of the mean values.

*Malignant transformation in M*2 *mouse fibroblasts.*—The M2 clone of mouse fibroblasts used to determine malignant transformation was originally obtained from C3H mouse prostate, and was established as a line by procedures described by Chen & Heidelberger (1969). This clone is susceptible to transformation by chemicals (Marquardt, 1973, 1976; Marquardt *et al.*, 1974). In the present work, cells were used between the 11th and 24th passages and the transformation assay was performed as previously reported (Marquardt *et al.*, 1974). In order to estimate plating efficiency and to assay transformation, $10^2$ and $10^3$ cells respectively were plated into 60mm dishes, and after 24 h the cultures were treated with freshly prepared solutions of the test compounds in dimethyl sulphoxide. After 24 h, the compounds were removed by changing the media; thereafter, the media was changed twice weekly. After 7–14 days, the cells in the dishes plated with $10^2$ cells were fixed and stained, and the colonies counted to determine plating efficiency. After 56 days, the dishes plated with $10^3$ cells were fixed, stained and scored for transformed, piled-up foci. The standard errors for the yields of transformed foci/$10^3$ plated cells ranged between 5% and 21% of the mean values.

In addition, in order to check the validity of this transformation assay, piled-up foci of morphologically transformed cells, areas of the same dish with normal morphology, and areas from control dishes were ring-isolated. The isolated cells were passaged twice and inoculated ($10^6$ cells) s.c. into inbred male C3H/HeJ mice that were observed for 6 months for tumour development.

### RESULTS AND DISCUSSION

Table I shows the results obtained when BA and 7,12-DMBA and some of their related dihydrodiols were tested for their ability to induce mutations to AZ resistance in V79 Chinese hamster cells and malignant transformations in M2 mouse fibroblasts. In the induction of mutations, the non-K-region 1,2- and 3,4-dihydrodiols of BA were active, whereas the K-region 5,6- and the non-K-region 8,9-dihydrodiols were inactive; the 10,11-dihydrodiol was not tested in this system

TABLE I.—*Mutagenesis and malignant transformation in mammalian cells by dihydrodiols derived from benz[a]anthracene (BA) and 7,12-dimethylbenz[a]anthracene (DMBA)**

| | | Mutagenesis in V79 cells† | | Transformation in M2 cells‡ | | |
|---|---|---|---|---|---|---|
| Compound | Concentration (μg/ml) | Plating efficiency (%) | AZ$^r$ colonies/$10^5$ survivors | Plating efficiency (%) | Transformed foci/dishes treated | Transformed foci/$10^3$ survivors |
| Dimethylsulphoxide§ | 0·5% | 84 | 2·7 | 31 | 0/9 | 0 |
| N-Methyl-N′-nitro-N-nitrosoguanidine§ | 0·4 | 31 | 294·1 | 21 | 10/9 | 5·3 |
| BA | 1·0 | 82 | 4·5 | 32 | 0/22 | 0 |
| | 10·0 | 70 | 5·4 | 29 | 8/22 | 1·3 |
| DMBA | 0·25 | 63 | 3·0 | 26 | 9/8 | 4·3 |
| | 1·0 | 39 | 4·4 | 24 | 19/11 | 7·2 |
| *trans*-1,2-Dihydro-1,2-dihydroxy BA** | 1·2 | 99 | 13·0 | 44 | 0/3 | 0 |
| | 2·5 | 95 | 16·6 | 40 | 0/12 | 0 |
| | 5·0 | 89 | 17·5 | 37 | 0/11 | 0 |
| | 10·0 | 55 | 41·5 | 10 | 1/19 | 0·5 |
| *trans*-3,4-Dihydro-3,4-dihydroxy BA | 2·5 | 93 | 16·4 | 50 | 0/10 | 0 |
| | 5·0 | 84 | 24·5 | 38 | 0/11 | 0 |
| | 10·0 | 56 | 56·5 | 12 | 0/12 | 0 |
| *trans*-3,4-Dihydro-3,4-dihydroxy-DMBA | 0·12 | 67 | 5·7 | 31 | 3/5 | 1·9 |
| | 0·25 | 58 | 19·8 | 26 | 9/10 | 3·5 |
| | 0·5 | 50 | 29·8 | 25 | 26/8 | 13·0 |
| | 1·0 | 43 | 44·1 | 15 | 20/9 | 14·3 |
| *trans*-5,6-Dihydro-5,6-dihydroxy BA | 2·5 | 85 | 2·6 | 44 | 0/12 | 0 |
| | 5·0 | 74 | 1·3 | 42 | 0/12 | 0 |
| | 10·0 | 59 | 0 | 40 | 0/12 | 0 |
| *trans*-5,6-Dihydro-5,6-dihydroxy-DMBA | 1·0 | 80 | 2·0 | 28 | 0/10 | 0 |
| | 5·0 | 73 | 0·9 | 28 | 0/10 | 0 |
| | 10·0 | 57 | 0 | 20 | 0/10 | 0 |
| *trans*-8,9-Dihydro-8,9-dihydroxy-BA | 2·5 | 57 | 1·5 | 44 | 0/12 | 0 |
| | 5·0 | 54 | 1·6 | 39 | 0/12 | 0 |
| | 10·0 | 53 | 1·6 | 30 | 0/12 | 0 |
| *trans*-8,9-Dihydro-8,9-dihydroxy-DMBA | 0·25 | 62 | 1·9 | 26 | 4/4 | 3·8 |
| | 0·5 | 45 | 20·5 | 22 | 15/5 | 13·6 |
| | 1·0 | 32 | 42·5 | 7 | 11/6 | 26·2 |
| *trans*-10,11-Dihydro-10,11-dihydroxy-BA | 2·5 | — | — | 44 | 0/12 | 0 |
| | 5·0 | — | — | 37 | 0/12 | 0 |
| | 10·0 | — | — | 29 | 0/12 | 0 |
| *trans*-10,11-Dihydro-10,11-dihydroxy-DMBA | 0·25 | 76 | 6·3 | 30 | 0/8 | 0 |
| | 0·5 | 68 | 5·6 | 26 | 0/12 | 0 |
| | 1·0 | 44 | 4·9 | 11 | 0/11 | 0 |

* Composite results from 2–4 separate experiments.
† Cells were grown in media containing the test compound for 3 h.
‡ Cells were grown in media containing the test compound for 24 h.
§ These data serve as controls for the experiments using DMBA or its derivatives. The control data for the experiments using BA and its derivatives are given in Table II.
** The corresponding 1,2-dihydrodiol of DMBA was not available for testing.

because of a shortage of material at the time the experiments were carried out. In contrast, the non-K-region 8,9-dihydrodiol of DMBA, as well as the corresponding 3,4-dihydrodiol, were active. The K-region 5,6-dihydrodiol and the non-K-region 10,11-dihydrodiol were inactive: the related 1,2-dihydrodiol was not available for testing. Both the parent hydrocarbons were inactive in this test system, presumably because of the low metabolic capabilities of V79 cells.

In the M2 cells, both parent hydrocarbons showed some activity, although BA was much less active than DMBA, even at the higher concentrations used for the former compound. None of the 5 dihydrodiols of BA showed significant activities in the induction of malignant transformation, whereas at a dose level of 1 μg/ml both the 3,4- and 8,9-dihydrodiols of DMBA appeared to be more active than the parent hydrocarbon, if the frequency of malignant transformation is considered in terms of the numbers of surviving cells. It should be noted, however, that in the experiments carried out with BA derivatives, the M2 cells showed unusually high plating efficiencies that were not apparent in the experiments performed later with the DMBA derivatives. The increased plating efficiencies were most probably due to a particular batch of foetal calf serum that supported the growth of the M2 cells especially well.

Morphologically transformed cells were also tested for their abilities to induce tumours in isologous, unirradiated mice. Five weeks after the injection into C3H/HeJ mice (3 mice/clone) of cells ($10^6$ cells) from 2 clones transformed by the 3,4-dihydrodiol derived from DMBA, all 6 animals had developed malignant fibrosarcomas as diagnosed by histological examination. The tumours did not metastasize. Six mice that received injections of cells from control cultures or of cells with normal morphology from treated dishes did not develop tumours.

The mutagenic activity shown by the 3,4-dihydrodiol of BA is in agreement with results of other studies, where the diol was active as a mutagen both in V79 cells (Slaga *et al.*, 1978) and in *Salmonella typhimurium* TA100 (Wood *et al.*, 1976) in the presence of mono-oxygenase systems. However, the mutagenic activity shown by the 1,2-dihydrodiol was unexpected since it was not found to be active by other workers either in V79 cells (Slaga *et al.*, 1978) or in *S. typhimurium* (Wood *et al.*, 1976). In the present work, however, the mutagenicity studies in V79 cells were carried out in the absence of the secondary cultures of embryo cells often used to augment the low metabolizing abilities of the V79 cells. The high mutagenic activity of the 3,4-dihydrodiol of DMBA is in accord with its high microsome-mediated mutagenic activity in *S. typhimurium* TA100 (Malaveille *et al.*, 1978). The high mutagenic activity shown by the 8,9-dihydrodiol of DMBA is perhaps unexpected, since the diol-epoxide formed from this diol by metabolism is not a vicinal bay-region diol-epoxide. However, the 8,9-dihydrodiol showed some mutagenic activity in *S. typhimurium* (Malaveille *et al.*, 1978) and the related 8,9-dihydrodiol of 7-methylbenz[a]anthracene was active in V79 cells (Marquardt *et al.*, 1977*b*).

The inability of the 3,4-dihydrodiol of BA to induce malignant transformation in M2 cells is surprising, since BA itself induced some transformations and the diol is highly active, both as an initiator of tumours on mouse skin (Wood *et al.*, 1977*b*; Slaga *et al.*, 1978) and as an inducer of tumours in newborn mice (Wislocki *et al.*, 1976). On the other hand, the 3,4-dihydrodiol of DMBA, which induces malignant transformation in M2 cells, also acts as an initiator of tumours on mouse skin (I. Chouroulinkov, personal communication). The 8,9-dihydrodiol of DMBA also induces malignant transformations in M2 cells, in agreement with earlier observations (Marquardt *et al.*, 1976), where both this diol and the 8,9-dihydrodiol of 7-methylbenz[a]anthracene were active in M2 cells. It is not yet known why these two diols are biologically active since neither compound can give rise to a bay-region diol-epoxide of the type on metabolism described by Jerina *et al.* (1976).

Table II shows the results of a short study on the biological activities of the 2 isomeric 8,9-dihydrodiol-10,11-epoxides of BA. In V79 cells, the *anti*-isomer was more active as a mutagen than the *syn*-isomer, and in M2 cells the *anti*-isomer was active in inducing malignant transformation, whilst the *syn*-isomer was inactive. Wood *et al.* (1977*a*) similarly showed that the *anti*-isomer of this diol-epoxide is more active than the *syn* in inducing mutations in V79 cells. The *anti*-isomer of the 7,8-dihydrodiol-9,10-epoxide of benzo[a]pyrene is also more active than

TABLE II.—*Mutagenesis and malignant transformation in mammalian cells by diol-epoxides of benz[a]anthracene BA**

| Compound | Concentration (μg/ml) | Mutagenesis in V79 cells† Plating efficiency (%) | Mutagenesis in V79 cells† AZ^r colonies/10^5 survivors | Transformation in M2 cells‡ Plating efficiency (%) | Transformation in M2 cells‡ Transformed foci/dishes treated | Transformation in M2 cells‡ Transformed foci/10^3 survivors |
|---|---|---|---|---|---|---|
| Dimethylsulphoxide§ | 0·5% | 95 | 9·9 | 51 | 0/29 | 0 |
| N-Methyl-N′-nitro-N- | 0·2 | 74 | 85·1 | 32 | 23/24 | 3·0 |
| nitrosoguanidine§ | 0·4 | 56 | 143·2 | 26 | 25/19 | 5·1 |
| (±)-8β, 9α-Dihydroxy-10β, | 0·6 | 56 | 44·1 | — | — | — |
| 11β-epoxy-8,9,10,11- | 1·2 | 50 | 69·9 | 47 | 0/6 | 0 |
| tetrahydro BA (*syn*- | 2·5 | 49 | 68·3 | 49 | 0/6 | 0 |
| isomer) | 5·0 | 48 | 69·4 | 30 | 0/6 | 0 |
| (±)-8β, 9α-Dihydroxy- | 0·6 | 57 | 116·7 | — | — | — |
| 10α,11α-epoxy-8,9,10,11- | 1·2 | 48 | 126·6 | 26 | 1/6 | 0·9 |
| tetrahydro BA (*anti*- | 2·5 | 41 | 156·0 | 12 | 4/6 | 5·6 |
| isomer) | 5·0 | 35 | 187·7 | 6 | 4/6 | 11·3 |

* Composite results from 2 separate experiments.
† Cells were grown in media containing the test compound for 3 h.
‡ Cells were grown in media containing the test compound for 24 h.
§ These data serve as controls for the experiments with BA and its derivatives shown in Table I.

the *syn* in inducing transformations in M2 cells (Marquardt *et al.*, 1977*a*). When the 8,9-dihydrodiol of BA is metabolized by a rat-liver microsomal fraction, a 8,9-dihydrodiol-10,11-oxide was detected as a metabolite (Booth & Sims, 1974); this oxide has now been shown to be the *anti*-isomer (P. Sims, unpublished observations).

BA and DMBA are metabolized by rat-liver microsomal fractions to a number of dihydrodiols (Tierney *et al.*, 1978*c*), the major products being the 5,6- and 8,9-dihydrodiols. Smaller amounts of the 10,11-dihydrodiols are formed from both hydrocarbons, but whereas BA yields a little 1,2-dihydrodiol, no 1,2-dihydrodiol is formed from DMBA. In contrast, some 3,4-dihydrodiol is formed from 7,12-DMBA but only trace amounts of the corresponding dihydrodiol from benz[a]anthracene. However, Levin *et al.* (1978) suggest that, with BA, an isomer of the 3,4-dihydrodiol-1,2-oxide is the ultimate carcinogen. The species derived from this hydrocarbon that binds to the DNA or hamster embryo cells was originally thought to be one of the isomers of the 8,9-dihydrodiol-10,11-epoxide tested here (Swaisland *et al.*, 1974), but this now seems less likely in view of more recent results.

The particular diol-epoxides derived from DMBA that are involved in DNA binding have not yet been identified with certainty, but fluorescence studies (Vigny *et al.*, 1977*b*; Moschel *et al.*, 1977; Ivanovic *et al.*, 1978) on the DNA of mouse skin or cells in culture have implicated products formed on the 1,2,3,4-ring of the hydrocarbon. Assuming that reactions with DNA are related to biological activity, the present results and some others (Malaveille *et al.*, 1978) suggest that these products are one or both of the isomers of the 3,4-dihydrodiol-1,2-epoxide.

The authors wish to thank Alan Hewer and Christine Walsh for excellent technical assistance. This work was supported by grants to the Chester Beatty Research Institute, Institute of Cancer Research: Royal Cancer Hospital from the Medical Research Council and the Cancer Research Campaign and by Grants No. CA-08748 and CA-15205 from the National Cancer Institute, USPHS to the Memorial Sloan–Kettering Cancer Center. H. Marquardt is the recipient of Research Career Development Award 1 KO4 CA 00127 from the National Institutes of Health, USPHS.

### REFERENCES

BAIRD, W. M. & BROOKES, P. (1973) Isolation of the hydrocarbon-deoxyribonucleoside products from the DNA of mouse embryo cells treated in culture with 7-methylbenz[a]anthracene-$^3$H. *Cancer Res.*, **33**, 2378.

BOOTH, J. & SIMS, P. (1974) 8,9-Dihydro-8,9-dihydroxybenz[a]anthracene 10, 11-oxide: a new type of polycyclic aromatic hydrocarbon metabolite. *FEBS Lett.*, **47**, 30.

BORGEN, A., DARVEY, H., CASTAGNOLI, N., CROCKER, T. T., RASMUSSEN, R. E. & WANG, I. Y. (1973) Metabolic conversion of benzo[a]pyrene by Syrian hamster liver microsomes and binding of metabolites to deoxyribonucleic acid. *J. Med. Chem.*, **16**, 502.

BOYLAND, E. & SIMS, P. (1964) Metabolism of polycyclic compounds 24. The metabolism of benz[a] anthracene. *Biochem. J.*, **91**, 493.

BOYLAND, E. & SIMS, P. (1965) The metabolism of 7,12-dimethylbenz[a]anthracene by rat liver homogenates. *Biochem. J.*, **95**, 780.

BOYLAND, E. & SIMS, P. (1967) The effect of pretreatment with adrenal protecting compounds on the metabolism of 7,12-dimethylbenz[a]anthracene and related compounds by rat-liver homogenates. *Biochem. J.*, **104**, 394.

CHEN, T. T. & HEIDELBERGER, C. (1969) Cultivation *in vitro* of cells derived from adult C3H mouse ventral prostate. *J. Natl Cancer Inst.*, **42**, 903.

CHOUROULINKOV, I., GENTIL, A., GROVER, P. L. & SIMS, P. (1976) Tumour initiating activities on mouse skin of dihydrodiols derived from benzo[a] pyrene. *Br. J. Cancer*, **34**, 523.

CHOUROULINKOV, I., GENTIL, A., TIERNEY, B., GROVER, P. L. & SIMS, P. (1977) The metabolic activation of 7-methylbenz[a]anthracene in mouse skin: high tumour initiating activity of the 3,4-dihydrodiol. *Cancer Lett.*, **3**, 247.

CHU, E. H. Y. & MALLING, H. V. (1968) Mammalian cell genetics II. Chemical induction of specific locus mutations in Chinese hamster cells *in vitro*. *Proc. Natl Acad. Sci. U.S.A.*, **61**, 1306.

DAUDEL, P., DUQUESNE, M., VIGNY, P., GROVER, P. L. & SIMS, P. (1975) Fluorescence spectral evidence that benzo[a]pyrene-DNA products in mouse skin arise from diol-epoxides. *FEBS Lett.*, **57**, 250.

HUBERMAN, E., ASPIRAS, L., HEIDELBERGER, C., GROVER, P. L. & SIMS, P. (1971) Mutagenicity to mammalian cells of epoxides and other derivatives of polycyclic hydrocarbons. *Proc. Natl Acad. Sci. U.S.A.*, **68**, 3195.

IVANOVIC, I., GEACINTOV, N. E., JEFFREY, A. M., FU, P. P., HARVEY, R. G. & WEINSTEIN, I. B. (1978) Cell and microsome mediated binding of 7,12-dimethylbenz[a]anthracene to DNA studied by fluorescence spectroscopy. *Cancer Lett.*, **4**, 131.

JEFFREY, A. M., WEINSTEIN, I. B., JENNETTE, K. W. & 5 others (1977) Structures of benzo[a]pyrene-nucleic-acid adducts formed in human and bovine bronchial explants. *Nature*, **269**, 348.

JERINA, D. M., LEHR, R. E., YAGI, H. & 7 others (1976) Mutagenicity of benzo[a]pyrene derivatives and the description of a quantum mechanical model which predicts the ease of carbonium ion formation from diolepoxides. In *In Vitro Metabolic Activation in Mutagenesis Testing*, Eds F. J. de Serres, J. R. Fouts, J. R. Bend and R. M. Philpot. Amsterdam: North Holland, p. 159.

KING, H. W. S., OSBORNE, M. R. & BROOKES, P. (1977) The metabolism and DNA binding of 3-methylcholanthrene. *Int. J. Cancer*, **20**, 564.

LEHR, R. E., SCHAEFER-RIDDER, M. & JERINA, D. M. (1977a) Synthesis and properties of the vicinal *trans*-dihydrodiols of anthracene, phenanthrene and benz[a]anthracene. *J. Org. Chem.*, **42**, 736.

LEHR, R. E., SCHAEFER-RIDDER, M. & JERINA, D. M. (1977*b*) Synthesis and reactivity of diolepoxides derived from non-K-region *trans*-dihydrodiols of benz[a]anthracene. *Tetrahedron Lett.*, 539.

LEVIN, W., THAKKER, D. R., WOOD, A. W. & 4 others (1978) Evidence that benzo[a]anthracene-3, 4-diol-1,2-epoxide is an ultimate carcinogen on mouse skin. *Cancer Res.*, **38**, 1705.

LEVIN, W., WOOD, A. W., YAGI, H., JERINA, D. M. & CONNEY, A. H. (1976) (±)-trans-7,8-Dihydroxy-7,8-dihydrobenzo[a]pyrene: A potent skin carcinogen when applied topically to mice. *Proc. Natl Acad. Sci. U.S.A.*, **73**, 3867.

MALAVEILLE, C., BARTSCH, H., GROVER, P. L. & SIMS, P. (1975) Mutagenicity of non-K-region diols and diol-epoxides of benz[a]anthracene and benzo-[a]pyrene in *S. typhimurium* TA100. *Biochem. Biophys. Res. Commun.*, **66**, 693.

MALAVEILLE, C., BARTSCH, H., TIERNEY, B., GROVER, P. L. & SIMS, P. (1978) Microsome-mediated mutagenicities of the dihydrodiols of 7,12-dimethylbenz[a]anthracene: high mutagenic activity of the 3,4-dihydrodiol. *Biochem. Biophys. Res. Commun.*, **83**, 1468.

MALAVEILLE, C., TIERNEY, B., GROVER, P. L., SIMS, P. & BARTSCH, H. (1977) High microsome-mediated mutagenicity of the 3,4-dihydrodiol of 7-methylbenz[a]anthracene in *S. typhimurium* TA98. *Biochem. Biophys. Res. Commun.*, **75**, 427.

MARQUARDT, H. (1973) The effect of X-irradiation on hydrocarbon metabolism and on hydrocarbon-induced lethality and transformation in cells derived from mouse prostate. *Z. Krebsforsch.*, **80**, 223.

MARQUARDT, H. (1976) Malignant transformation *in vitro*: A model system to study mechanisms of action of chemical carcinogens and to evaluate the oncogenic potential of environmental chemicals. In *Screening Tests in Chemical Carcinogenesis*, Eds R. Montesano, H. Bartsch and L. Tomatis. Lyon. International Agency for Research on Cancer. p: 389.

MARQUARDT, H., BAKER, S., GROVER, P. L. & SIMS, P. (1977*a*) Malignant transformation and mutagenesis in mammalian cells induced by vicinal diol-epoxides from benzo[a]pyrene. *Cancer Lett.*, **3**, 31

MARQUARDT, H., BAKER, S., TIERNEY, B., GROVER, P. L. & SIMS, P. (1977*b*) The metabolic activation of 7-methylbenz[a]anthracene: The induction of malignant transformation and mutation in mammalian cells by non-K-region dihydrodiols. *Inst. J. Cancer*, **19**, 828.

MARQUARDT, H., GROVER, P. L. & SIMS, P. (1976) *In vitro* malignant transformation of mouse fibroblasts by non-K-region dihydrodiols derived from 7-methylbenz[a]anthracene, 7,12-dimethylbenz[a]anthracene and benzo[a]pyrene *Cancer Res.*, **36**, 2059.

MARQUARDT, H., SODERGREN, J. E., SIMS, P. & GROVER, P. L. (1974) Malignant transformation *in vitro* of mouse fibroblasts by 7,12-dimethylbenz[a]anthracene and 7-hydroxymethylbenz-[a]anthracene and by their K-region derivatives. *Int. J. Cancer*, **13**, 304.

MOSCHEL, R. C., BAIRD, W. M. & DIPPLE, A. (1977) Metabolic activation of the carcinogen 7,12-dimethylbenz[a]anthracene for DNA binding. *Biochem. Biophys. Res. Commun.*, **76**, 1092.

SIMS, P. (1970) Quantitative and qualitative studies on the metabolism of a series of aromatic hydrocarbons by rat-liver preparations. *Biochem. Pharmacol.*, **19**, 795.

SIMS, P., GROVER, P. L., SWAISLAND, A., PAL, K. & HEWER, A. (1974) Metabolic activation of benzo-[a]pyrene proceeds by a diol-epoxide. *Nature*, **252**, 326.

SLAGA, T. J., HUBERMAN, E., SELKIRK, J. K., HARVEY, R. G. & BRACKEN, W. M. (1978) Carcinogenicity and mutagenicity of benz[a]anthracene diols and diol-epoxides. *Cancer Res.*, **36**, 1699.

SLAGA, T. J., VIAJE, A., BERRY, D. L., BRACKEN, W., BUTY, S. G. & SCRIBNER, J. D. (1966) Skin tumour initiating ability of benzo[a]pyrene 4,5-, 7,8- and 7,8-diol-9,10-epoxides and 7,8-diol. *Cancer Lett.*, **2**, 115.

SWAISLAND, A. J., HEWER, A., PAL, K., KEYSELL, G. R., BOOTH, J., GROVER, P. L. & SIMS, P. (1974) Polycyclic hydrocarbon epoxides: The involvement of 8,9-dihydro-8,9-dihydroxybenz[a]anthracene 10,11-oxide in reactions with the DNA of benz[a]anthracene-treated hamster embryo cells. *FEBS Lett.*, **17**, 34.

THAKKER, D. R., LEVIN, W., WOOD, A. W., CONNEY, A. H., STOMING, T. A. & JERINA, D. M. (1978) Metabolic formation of 1,9,10-trihydroxy-9,10-dihydro-3-methylcholanthrene: A potential proximate carcinogen from 3-methylcholanthrene. *J. Am. Chem. Soc.*, **100**, 645.

TIERNEY, B., ABERCROMBIE, B., WALSH, C., HEWER, A., GROVER, P. L. & SIMS, P. (1978*a*) The preparation of dihydrodiols from 7-methylbenz[a]anthracene. *Chem.-Biol. Interact.*, **21**, 289.

TIERNEY, B., HEWER, A., MACNICOLL, A. D. & 5 others (1978*b*) The formation of dihydrodiols by the chemical and enzymic oxidation of benz[a]-anthracene and 7,12-dimethylbenz[a]anthracene. *Chem.-Biol. Interact.*, **23**, 243.

TIERNEY, B., HEWER, A., RATTLE, H., GROVER, P. L. & SIMS, P. (1978*c*) The formation of dihydrodiols by chemical or enzymic oxidation of 3-methylcholanthrene. *Chem.-Biol. Interact.*, **23**, 121.

TIERNEY, B., HEWER, A., WALSH, C., GROVER, P. L. & SIMS, P. (1977) The metabolic activation of 7-methylbenz[a]anthracene in mouse skin. *Chem.-Biol. Interact.*, **18**, 179.

VIGNY, P., DUQUESNE, M., COULOMB, H. & 4 others (1977*a*) Metabolic activation of polycyclic hydrocarbons. Fluorescence spectral evidence is consistent with metabolism at the 1,2- and 3,4-double bonds of 7-methylbenz[a]anthracene. *FEBS Lett.*, **75**, 9.

VIGNY, P., DUQUESNE, M., COULOMB, H., TIERNEY, B., GROVER, P. L. & SIMS, P. (1977*b*) Fluorescence spectral studies on the metabolic activation of 3-methylcholanthrene and 7,12-dimethylbenz[a]-anthracene in mouse skin. *FEBS Lett.*, **82**, 278.

WISLOCKI, P. G., WOOD, A. W., CHANG, R. L. & 6 others (1976) Mutagenicity and cytotoxicity of benzo[a]pyrene arene oxides, phenols, quinones and dihydrodiols in bacteria and mammalian cells. *Cancer Res.*, **36**, 3350.

WOOD, A. W., CHANG, R. L., LEVIN, W. & 5 others (1977*a*) Mutagenicity and cytotoxicity of benz]a[-anthracene diol epoxides and tetrahydro-epoxides: Exceptional activity of the bay region 1,2-epoxides. *Proc. Natl Acad. Sci. U.S.A.*, **74**, 2746.

WOOD, A. W., LEVIN, W., CHANG, R. L. & 5 others (1977*b*) Tumorigenicity of five dihydrodiols of benz[a]anthracene on mouse skin: Exceptional activity of benz[a]anthracene 3,4-dihydrodiol. *Proc. Natl Acad. Sci. U.S.A.*, **74**, 3176.

WOOD, A. W., LEVIN, W., LU, A. Y. & 6 others (1976) Mutagenicity of metabolically activated benz[a]anthracene 3,4-dihydrodiol: Evidence for bay region activation of carcinogenic polycyclic hydrocarbons. *Biochem. Biophys. Res. Commun.*, **72**, 680.